# The Role of Magnesium, Zinc, and Strontium in Osteoporotic Fracture Repair

**DOI:** 10.3390/bioengineering12020201

**Published:** 2025-02-18

**Authors:** Zhen Wang, Penghui Xiang, Zhe Xu, Meiqi Gu, Rui Zhang, Yifei Li, Hua Chen, Li He, Chengla Yi

**Affiliations:** 1 Department of Traumatic Surgery, Tongji Hospital, Tongji Medical College, Huazhong University of Science and Technology, Wuhan 430030, China; wzhen_wyc@163.com (Z.W.);; 2 Department of Orthopaedic Trauma, Chinese PLA General Hospital (301 Hospital), Beijing 100853, China

**Keywords:** osteoporotic fractures, magnesium, zinc, strontium

## Abstract

Osteoporotic fractures represent a significant public health challenge in the context of an aging global population, with the rising prevalence of osteoporosis intensifying the demand for effective fracture treatment. Restoring the structure and function of bone tissue damaged by osteoporosis-induced defects remains a critical issue in clinical practice. In recent years, bioactive metallic materials such as magnesium, zinc, and strontium have gained considerable attention due to their exceptional mechanical properties, biocompatibility, and biodegradability, positioning them as promising materials for osteoporotic fracture repair. This review systematically explored the biological mechanisms, application advancements, and associated challenges of magnesium, zinc, and strontium in fracture healing. Key topics included their roles in promoting osteoblast proliferation and differentiation, inhibiting osteoclast activity, and modulating the bone microenvironment. Additionally, this review examined the optimization strategies for their clinical application, such as their integration into bone scaffolds, the functionalization of conventional materials, and the synergistic effects between different metals. Finally, this review analyzed the current progress and unresolved issues in this field, offering a forward-looking perspective on the clinical potential of bioactive metallic materials in precision treatment of osteoporotic fractures.

## 1. Introduction

Osteoporotic fractures represent a global health challenge, particularly affecting the elderly, including postmenopausal women and older men [1,2]. The primary characteristics of osteoporotic fractures include a significant decrease in bone mineral density and the degradation of the trabecular bone structure, leading to fragile bones and difficulty healing [3]. According to global disease burden studies, millions of cases occur annually, posing a significant burden on patients’ quality of life and healthcare resources [4]. Addressing these fractures effectively remains a critical challenge in both clinical medicine and biomaterial research.

The traditional treatments for osteoporotic fractures, such as surgical internal fixation, hip replacement, and pharmacological therapies (e.g., bisphosphonates and recombinant human parathyroid hormone), can alleviate symptoms to some extent [5,6]. However, osteoporosis significantly alters the structural and biological properties of the bone, leading to increased fragility, reduced bone quality, and slower healing rates. These changes heighten the risk of complications, such as fixation loosening, implant fractures, and nonunion [7]. Specifically, the reduced bone mineral density and degradation of the trabecular structure compromise the anchoring strength of traditional implants, while an impaired regenerative capacity challenges effective long-term outcomes. Thus, traditional osteosynthesis devices face several challenges in osteoporotic bone. For example, screws and plates may lose their fixation strength in the weakened bone matrix, particularly under mechanical loading. This often results in implant migration, instability, or failure. These challenges highlight the need for innovative treatment strategies that address not only the mechanical support but also the biological deficiencies of osteoporotic bone.

In recent years, bioactive metallic materials such as magnesium, zinc, and strontium have emerged as key materials in the treatment of osteoporotic fractures due to their outstanding mechanical properties, osteoinductive potential, anti-resorptive capabilities, and high biocompatibility [8,9,10]. They promote bone healing through mechanisms such as osteoblast differentiation, osteoclast inhibition, enhanced angiogenesis, and local microenvironment regulation [11,12]. For instance, strontium ions (Sr^2+^) enhance osteoblast activity and suppress osteoclast function via the RANKL/OPG pathway, contributing to the dynamic balance between bone resorption and formation [13]. The magnesium ions (Mg^2+^) released during degradation regulate the differentiation of bone marrow stromal cells. Additionally, its mechanical properties, which closely resemble those of natural bone tissue, make magnesium an ideal material for load-bearing applications [14]. Zinc (Zn) and copper (Cu) ions promote angiogenesis, the formation of new blood vessels, thereby enhancing blood supply and supporting the bone healing process [15,16].

**Figure 1:**
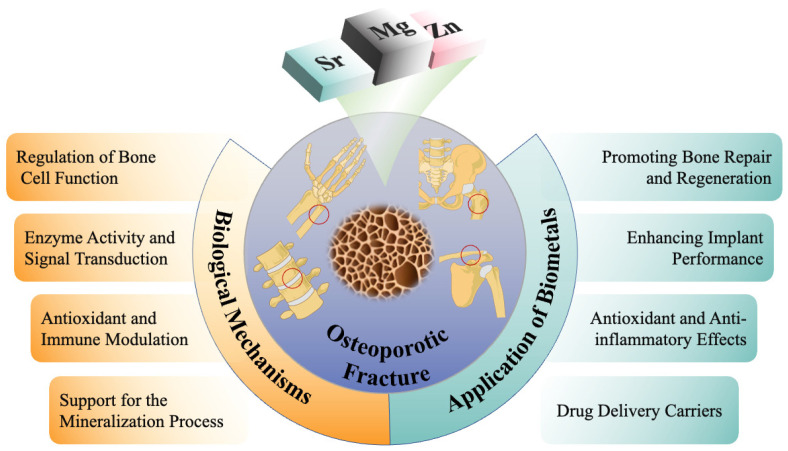
Mechanism and application of metals in osteoporotic fractures.

Despite advances, the existing reviews often emphasize general overviews of osteoporotic fractures or drug therapies, lacking a systematic examination of bioactive metallic materials [17,18,19,20]. Therefore, this review focuses on the recent progress in the use of magnesium, zinc, and strontium for osteoporotic fracture repair. It explores their biological mechanisms and applications, strategies for performance optimization, the current challenges, and future prospects, providing insights for advancing this field (Figure 1).

## 2. Materials and Methods

This study presents an analytical review of magnesium, zinc, and strontium related to osteoporotic fractures, providing a detailed explanation of their biological mechanisms and applications in osteoporotic fractures. Data were collected in November 2024 using the following databases—PubMed, WOS, Elsevier, Wiley Online Library, Springer Nature, American Chemical Society, and Google Scholar—as well as the scientific article and patent databases “The LENS” and “ORBIT Intelligence”. The selection of these databases was based on their relevance and the comprehensiveness of their coverage in the field of biomedical research. PubMed and WoS are widely regarded for their extensive collection of peer-reviewed articles, while Elsevier, Wiley Online Library, Springer Nature, and the American Chemical Society provide access to a variety of high-quality journals. Google Scholar served as a supplementary source, allowing for the inclusion of gray literature and less mainstream studies. Scientific articles and patent databases, such as “The LENS” and “ORBIT Intelligence”, were incorporated to ensure a broad and thorough search of both academic publications and novel innovations in the field.

The inclusion criteria for this work consisted of original articles specifically related to osteoporotic fractures and metallic materials with their full texts available in Portuguese, English, and other languages. The exclusion criteria included abstracts, non-scientific websites, incomplete texts, and irrelevant or duplicate articles. The descriptive terms used in this study included (magnesium or Mg or strontium or Sr or zinc or Zn or metal) and (osteoporotic fractures). Articles were selected by reading the publication titles and abstracts related to the Boolean descriptors “OR” and “AND” to optimize the sample.

This review primarily draws upon 51 articles published after 2019. However, some older articles are also referenced to provide relevant background or verifiable information. This study suggests that the potential and development of magnesium, zinc, and strontium in the treatment of osteoporotic fractures warrant further investigation.

## 3. Mechanisms of Metals in the Treatment of Osteoporotic Fractures

The role of biometals in fracture repair depends not only on their physical properties but also on the biological effects of the ions they release in the bone microenvironment. Biometals such as Sr, Mg, Zn, Fe, and Cu play a key role in fracture repair through a series of complex biological mechanisms. They not only regulate cell proliferation, differentiation, and function but also, through specific molecular signaling pathways, modulate the local microenvironment, angiogenesis, and immune responses, promoting bone tissue regeneration and repair, demonstrating diverse repair potential.

### 3.1. Magnesium

**Figure 2:**
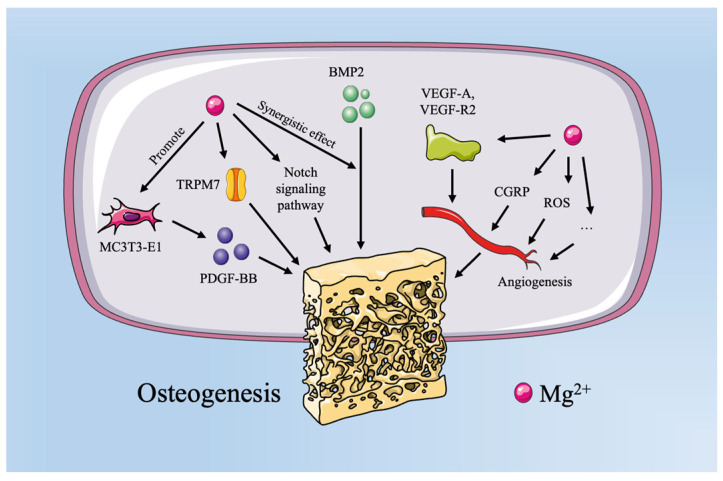
Mechanism of Mg^2+^ in osteoporotic fractures.

Mg^2+^ is the fourth most abundant element in the human body and is essential for the formation of soft tissues and bones [21]. Most of the Mg^2+^ in the body is stored in the bone tissue, where it promotes bone formation and angiogenesis and can be applied in the treatment of various types of bone defects [22]. A decrease in the Mg^2+^ ion content in the bone tissue weakens the mechanical properties of bone, causing trabecular fractures and increasing bone fragility and the risk of fractures [23] (Figure 2).

Mg^2+^ is an important osteogenic regulator that plays roles in both promoting osteoblastic differentiation and inhibiting osteoclast function and bone resorption. On the one hand, Mg^2+^ can activate the Notch signaling pathway to enhance the expression of osteogenic-related mRNA (such as alkaline phosphatase (ALP), RUNX family transcription factor-2 (Runx-2), and osteocalcin), thereby promoting osteoblast differentiation [24,25]. Liu et al. found that Mg^2+^ promoted the expression of mouse embryonic osteoblast precursor cells (MC3T3-E1) and the secretion of platelet-derived growth factor (PDGF), thus enhancing osteoblast differentiation and promoting angiogenesis in endothelial cells [26]. On the other hand, Wu et al.’s study demonstrated that a cell culture medium containing Mg^2+^ at certain concentrations exhibited strong toxicity to osteoclasts in monoculture, inhibiting osteoclast differentiation [27].

In addition, Mg^2+^ plays an important role in promoting angiogenesis and regulating bone immunity [28]. Gu et al. found that human bone marrow mesenchymal stem cells (hBMSCs) and human umbilical vein endothelial cells (HUVECs) exhibited high proliferation rates, good cell morphology, and viability in the presence of a certain amount of Mg^2+^, along with increased expression of osteogenic and angiogenic biomarkers [29]. Further studies by Li et al. indicated that Mg-Zn-Mn alloy extracts induce angiogenesis in human umbilical vein endothelial cells through the FGF/FGFR signaling pathway [30]. Recent in vivo studies have revealed that Mg^2+^⁺ plays a dynamic immunomodulatory role by influencing macrophage polarization across different phases of tissue repair. Initially, magnesium implants and the Mg^2+^ ions they release are associated with transient activation of the M1 macrophage phenotype, which is essential for initiating the early inflammatory phase required for tissue repair and implant integration. This early-phase M1 polarization contributes to the release of pro-inflammatory cytokines such as TNF-α and IL-6, which recruit additional immune cells and promote the removal of debris at the injury site. Following this, the inflammatory response resolves as Mg^2+^ induces a shift toward M2 macrophage polarization, which is marked by the increased secretion of anti-inflammatory cytokines like IL-10 and IL-1ra. The M2 phenotype supports angiogenesis, modulates osteogenesis, and contributes to tissue remodeling [31,32,33]. For instance, Liu et al. reported that Mg-based implants promoted this M1-to-M2 transition in a rat femoral defect model, contributing to enhanced osteogenesis and reduced inflammatory responses [34]. These findings underscore the immunomodulatory properties of Mg^2+^ in balancing inflammatory and regenerative phases, providing a more nuanced understanding of its role in the bone repair process.

### 3.2. Zinc

Zinc is also a relatively abundant trace element in the human body, and several metalloproteins in the body are regulated and catalyzed by Zn^2+^, such as alkaline phosphatase, which is closely related to new bone maturation [35]. During bone healing, Zn^2+^ mainly exerts various functions by regulating osteoblasts, osteoclasts, endothelial cells, and immune cells, including promoting the osteogenic differentiation of mesenchymal stem cells (MSCs), enhancing osteoblast bone formation, inhibiting osteoclast bone resorption, stimulating angiogenesis, and inducing immune regulation to promote new bone formation [36,37,38].

Zn^2+^-mediated osteogenic differentiation is dose-dependent. Yu et al. showed that low concentrations of Zn^2+^ (2–5 μg/mL) can enhance the initial adhesion and proliferation of BMSCs and subsequently regulate Zn^2+^ transport to induce osteogenic differentiation. In contrast, excessively high concentrations of Zn^2+^ (5 μg/mL) reduce cell adhesion and proliferation and inhibit subsequent osteogenic differentiation due to the resulting Zn^2+^ homeostasis imbalance [39]. Cho et al. pointed out that Zn^2+^ deficiency in osteoblasts leads to Smad-1 activation and the downregulation of RUNX2 expression through the BMP-2 signaling pathway, inhibiting osteoblast differentiation. As the Zn^2+^ levels increase, Zn ions promote RUNX2 expression through BMP-2 signaling, promoting osteoblast differentiation [40]. Additionally, Park et al. reported that Zn^2+^ exerts an inhibitory effect on osteoclastogenesis through the Ca^2+^–calmodulin–phosphatase–NFATc1 signaling pathway (a major transcriptional regulator of osteoclastogenesis) [41].

### 3.3. Strontium

Strontium is a trace element in the human body and an important component of the bone tissue. It has a bidirectional regulatory effect, promoting bone formation and inhibiting bone resorption. Sr^2+^ can induce the osteogenic differentiation of mesenchymal stem cells (MSCs). Zhao et al. found that collagen hydroxyapatite material containing Sr^2+^ expressed higher β-catenin protein levels and significantly increased bone density compared to its non-Sr counterpart, indicating that Sr^2+^ can promote the osteogenic differentiation of MSCs via the Wnt/β-catenin signaling pathway [42]. Additionally, Atkins et al. cultured primary human osteoblasts in Sr-containing medium and found that Sr^2+^ could promote OPG expression, inhibit RANKL expression, and suppress osteoclast differentiation [43].

Additionally, Sr^2+^ can promote endothelial cell proliferation and vascularization. Gu et al. seeded human umbilical vein endothelial cells and MG63 cells onto Sr-doped tricalcium phosphate, tricalcium phosphate, and hydroxyapatite scaffolds and found that the cells on the Sr-doped tricalcium phosphate scaffolds showed better adhesion and spreading and significantly improved proliferation. Further studies revealed that the expression of vascular endothelial growth factor (VEGF) and basic fibroblast growth factor (bFGF) was significantly higher in the Sr-doped tricalcium phosphate group compared to that in the other two groups (*p* < 0.05), indicating that strontium-doped tricalcium phosphate could upregulate VEGF and bFGF protein levels [44]. Zhu et al.’s study also showed that strontium can promote angiogenesis by increasing the expression of vascular endothelial growth factor (VEGF) and fibroblast growth factor 2 (FGF2) [45].

## 4. The Application of Metals in the Treatment of Osteoporotic Fractures

### 4.1. Magnesium

Due to its excellent biomechanical properties and significant biocompatibility, Mg has been widely used in the treatment of osteoporotic fractures. On the one hand, it can serve as a scaffold or be combined with other materials as a supporting framework, and on the other hand, Mg can be used as a coating to modify other materials [46].

#### 4.1.1. Magnesium-Based Implants

Magnesium, with a bone-like density and elastic modulus, is a biodegradable material ideal for temporary implants in osteoporotic fractures, reducing stress shielding and eliminating the need for removal surgery.

As a cornerstone of osteoporosis management, bisphosphonate treatment plays a critical role in modulating bone remodeling. However, their prolonged use may excessively suppress bone turnover, leading to impaired fracture healing—particularly in atypical femoral fractures (AFFs) characterized by delayed union and fibrous tissue accumulation. Emerging evidence indicates that bisphosphonates such as zoledronic acid not only reduce serum levels of bone remodeling markers but also downregulate neuropeptide signaling pathways critical for fracture repair. Their biological impact extends to the bone–implant interface, where suppressed angiogenesis and osteoclast activity may compromise osseointegration [47]. A landmark study by Zheng et al. demonstrated that chronic bisphosphonate exposure suppresses calcitonin-gene-related peptide (CGRP) expression, redirecting myeloid progenitor cells toward an ECM-secreting fibroblast phenotype. This aberrant fibrogenesis creates a mechanical barrier at the fracture gap, preventing callus bridging—a pathological feature phenocopied in both clinical AFF specimens and preclinical models. To address this, Zheng et al. proposed an innovative magnesium-containing intramedullary nail (Mg-IMN) that stimulated endogenous CGRP release, effectively reversing bisphosphonate-induced fibrosis and restoring the fracture union rates to near-normal levels [48]. This approach capitalizes on magnesium’s dual role as a biodegradable implant material and a CGRP modulator, offering a paradigm shift in AFF management. Moreover, this study provided new insights into the potential of combining the Mg-IMN with systemic CGRP agonists or neuromodulatory therapies to enhance the therapeutic outcomes. Wan et al. combined biodegradable Mg-based implants with bisphosphonates, proposing a novel one-step electrodeposition method for drug-loaded coatings, enabling the gentle and sustained release of bisphosphonate drugs for the treatment of osteoporotic fractures [10]. In practical applications, Mg-based implants experience complex stresses during their implantation, leading to stress-induced corrosion and early damage or dissolution of the implant, resulting in a rapid Mg^2+^ degradation rate. Zhang et al. used metal corrosion inhibitors, such as phytic acid and zoledronic acid (a third-generation bisphosphonate), and applied them using a simple one-step immersion method, which showed enhanced proliferation of pre-osteoblasts (MC3T3-E1) and significantly inhibited the proliferation and differentiation of osteoclasts [49]. Huang et al. developed a Sr-doped hydroxyapatite (Sr-HA) coating with higher solubility for the surface of Mg alloys to slow down the corrosion of Mg-based materials [50]. Liu et al. combined melatonin with silane coupling agents on Mg and coated the surface with a poly (lactic acid-co-glycolic acid) copolymer to accelerate the release of melatonin and Mg ions, developing a drug/ion delivery system for the treatment of osteoporotic fractures [51]. Another issue with Mg alloys in application is that the magnesium implant can initiate abnormal activation of the osteoclasts and regulate sensory innervation of the bone healing tissue, leading to postoperative pain during the continuous stages of osteoporotic fracture healing [52]. Therefore, Qi et al. used a SrHPO_4_ coating to alleviate pain by upregulating the secretion of AICAR from bone-marrow-derived macrophages or osteoclast precursors [53].

Bone cement is commonly used as a filling material in orthopedics. However, its application and effectiveness are greatly limited due to its lack of bioactivity and high elastic modulus. Therefore, incorporating Mg as an additive to improve its performance is an effective approach. Liu et al. prepared novel bone cement by incorporating tetra calcium phosphate and Mg^2+^-containing Whitlockite into PMMA cement [54]. Zhu et al. integrated Pickering foam technology into a MgO-MgCl_2_-H_2_O reaction system, using MgO as an interface stabilizer to develop a 3D porous scaffold for treating osteoporotic fractures [55]. Further research by He et al. constructed a drug delivery system composed of chitosan and liquid-phase teriparatide solution, which was incorporated into Mg phosphate cement (CHI-TR MPC). The new material exhibited an almost neutral pH, a longer setting time, excellent injectability, enhanced compressive strength, and increased porosity. At the same time, the CHI-TR MPC scaffold facilitated the controlled release of ions and drugs, promoted osteogenic differentiation and mineralization through its abundant porous structure, and displayed an appropriate degradation rate [56].

#### 4.1.2. Magnesium Nanoparticle Coatings

Mg-based nanoparticle coatings have the potential to promote osteoporotic bone healing [57]. Chen et al. synthesized a series of Mg-Ga layered double hydroxide nanosheets on the surface of titanium implants treated with alkaline heat. They found that these coatings could form a suitable alkaline microenvironment while significantly enhancing the autophagic activity of mesenchymal stem cells (MSCs), inducing osteogenic differentiation, and inhibiting osteoclast genesis, showing potential applications in osteoporosis treatment [58]. Wang et al. modified Mg and the drug icariin into a 3D-printed porous Ti6Al4V scaffold. They found that the controlled release of icariin and Mg^2+^ in the biofunctionalized PT could significantly improve the polarization of M0 macrophages to the M2 type by inhibiting the Notch1 signaling pathway and inducing the secretion of anti-inflammatory cytokines, thus enhancing the bone integration between the scaffold and the osteoporotic bone. For osteoporotic bone defects with irregular geometries caused by fractures, metal–organic frameworks formed of metal ions interconnected by organic ligands are increasingly being used [59]. Luo et al. incorporated Mg chloride and gallic acid into gelatin microspheres to form Gel@Mg-MOF composite microspheres. On the one hand, they utilized the biocompatibility, controlled release, and biodegradability of the gelatin, while on the other hand, the release of Mg^2+^ promoted the osteogenic activity in RBMSCs and enhanced the angiogenic potential in HUVECs [60].

In conclusion, the multifunctionality and broad application prospects of Mg-based materials in the treatment of osteoporotic fractures have been widely recognized (Table 1). Through modification and functional design, further optimization of their biological properties has been achieved, providing more innovative solutions for the repair of osteoporotic fractures.

### 4.2. Zinc

As a degradable metal, Zn has a corrosion rate second only to that of Mg. However, the release rate of Zn^2+^ may be too high, potentially inhibiting endothelial cell activity and thereby affecting angiogenesis and osteogenesis [65]. In bone tissue engineering, Zn can be used as an implant or incorporated into bioceramics, calcium phosphate bone cement, and other polymers [66,67].

#### 4.2.1. Zinc-Based Implants

Zinc itself has relatively low mechanical properties and cytotoxicity, which makes it unable to meet the ideal standards for biodegradable metals. Zhang et al. were the first to report an in vivo study of Zn alloys in osteoporotic fracture repair [68]. They used Li and Sr to improve the mechanical properties and biological safety of Zn alloy, showing significant osteogenic induction and osteoporotic fracture healing effects. Ji et al. introduced Cu^2+^ and zirconium into Zn-based alloys and found that in an elderly osteoporotic rat model, these alloys could effectively promote fracture healing, demonstrating their potential to influence macrophage immune regulation and aid in bone repair for elderly osteoporotic patients [8].

#### 4.2.2. Zinc-Based Nanoparticle Coatings

Compared to functionalizing Zn itself, Zn^2+^ can be incorporated into bioactive glasses, bioceramics, calcium phosphate bone cement, and various other bioactive polymers to improve the properties of these materials. Mughal et al. used 3D-printed scaffolds made from polyetheretherketone (PEEK) and sodium carboxymethyl cellulose (Na-CMC) and functionalized the surface with Zn- and manganese-doped bioactive glass nanoparticles (Zn–Mn MBGNs). This demonstrated significant angiogenesis and osteoporotic fracture healing potential [69]. Guo et al. prepared Zn on silicon carbide nanoparticles and experimentally showed that the Zn-loaded SiC particles could serve as promising wound healing agents, promoting healthy bone formation in osteoporotic femoral fractures [70]. Luengo-Alonso et al. used bioactive glass rich in ZnO and loaded with osteogenic peptides such as osteostatin to treat osteoporotic bone defects. This significantly improved the bone formation and regeneration in rabbit osteoporotic bone models, demonstrating its potential in treating osteoporotic bone defects [71].

### 4.3. Strontium

Unlike Mg and Zn, Sr’s physical properties are less suitable for use as a supportive scaffold in the treatment of osteoporosis [72]. In recent years, research on the application of Sr has deepened, particularly in the doping of bone cement and the development of functional scaffold coatings. The introduction of Sr not only significantly enhances the biological activity of materials but also plays a crucial role in the mechanical optimization of bone repair [73].

#### 4.3.1. Strontium-Based Implants

The bone bioactivity of Sr makes it an ideal bone substitute material. Ca-Si-based bioceramics have shown an enhanced bone formation ability under normal conditions. Wu et al. further incorporated Sr into bioceramics and confirmed that it could increase the expression of osteogenesis and angiogenesis markers in osteoporotic bone marrow stromal cells (OVX BMSCs), which provided new insights for the treatment of bone defects in osteoporotic patients [74]. Zheng et al. prepared a Sr-substituted calcium phosphate silicate bioactive ceramic (Sr-CPS) and found that Sr-CPS extracts can promote osteogenesis by upregulating the Wnt/β-catenin signaling pathway. They also inhibited osteoclast genesis by downregulating the NF-κB signaling pathway, which demonstrated that Sr-CPS ceramics can dually regulate bone formation and resorption [75]. Similarly, Lee et al. introduced Sr-releasing nanoscale cement, which achieved the dual therapeutic effects of promoting osteogenesis and inhibiting osteoclast genesis [76]. To simultaneously deliver therapeutic drugs, Corvaglia et al. added Sr and a recombinant protein (i.e., ICOS-Fc) that inhibited osteoclast activity to calcium sulfate powder. This system stimulated bone regeneration while releasing biomolecules capable of limiting bone resorption for the treatment of vertebral compression fractures [9].

#### 4.3.2. Strontium Nanoparticle Coatings

Strontium nanoparticle coatings are commonly applied to the surface of metal implants to enhance the bone-inducing ability of the material. For example, Ge et al. loaded SR onto optimized POFC/β-TCP porous scaffolds using 3D printing technology. The experiments showed that POFC/β-TCP scaffolds loaded with Sr^2+^ ranelate enhanced the proliferation and differentiation of rBMSCs in osteogenesis, guiding bone regeneration [77]. Yuan et al. developed a biomimetic polyetherketone scaffold and functionalized it with Sr nanohydroxyapatite coatings. This promoted osteoporotic bone regeneration and delayed the adjacent bone loss [78]. Huang et al. used high-solubility Sr-doped hydroxyapatite (Sr-HA) coatings for Mg alloy scaffolds. Their study found that the Sr-HA coating not only acted as a physical barrier to delay the formation of Mg pitting corrosion but also provided Sr ions to seal the cracks induced during tensile deformation [50]. Katunar et al. functionalized titanium implants with Sr-doped bioactive glass particles. The implants showed good in vivo performance, and after 30 days of implantation, new mineralized bone appeared around the implants coated with Sr [79].

In summary, Sr, by being doped into traditional materials such as bone cement, promotes bone formation and inhibits bone resorption, demonstrating good bone-regulating effects. Strontium nanoparticle coatings enhance the bone-inducing ability, corrosion resistance, and biocompatibility of materials (Table 2). These studies provide important support for the optimization of materials for the repair of osteoporotic fractures.

## 5. Comparison of Mg, Zn, and Sr in Osteoporotic Fracture Repair

Mg, Zn, and Sr each possess distinct properties that make them suitable for specific applications in osteoporotic fracture repair depending on the clinical scenario and therapeutic needs.

Mg demonstrates exceptional biomechanical properties, closely matching human bone in its density and elastic modulus. This compatibility reduces stress shielding and supports its use as a biodegradable material for temporary implants. Additionally, Mg can promote osteogenesis and angiogenesis, offering significant benefits for load-bearing applications. However, challenges such as rapid degradation and stress-induced corrosion require careful material design to optimize its performance. Mg-based materials are particularly advantageous in clinical contexts demanding temporary support and mechanical stability during the healing process. Moreover, studies have shown that osteoporotic bone exhibits accelerated resorption rates compared to those in healthy bone, which can undermine the longevity and stability of biodegradable implants [89]. This phenomenon is particularly relevant for magnesium implants, as their degradation kinetics may be significantly influenced by the resorptive environment of osteoporotic bone. The study by Sommer et al. specifically highlights these differences, demonstrating that magnesium implants may degrade more rapidly in osteoporotic bone compared to healthy bone [61]. This necessitates further research to optimize the degradation behavior of Mg-based implants, ensuring their mechanical integrity and functional longevity in osteoporotic fracture repair.

Zinc exhibits moderate corrosion rates and plays a vital role in promoting angiogenesis and osteogenesis. Its incorporation into bioactive glasses, ceramics, and coatings enhances these materials’ biological performance. However, its relatively low mechanical strength and the cytotoxicity risk associated with high Zn ion concentrations limit its application in load-bearing environments. Zn-based materials are better suited to promoting vascularization and immune modulation, making them ideal for non-load-bearing areas or the treatment of bone defects with a focus on enhanced bone healing.

Strontium is distinguished by its ability to regulate bone metabolism, simultaneously enhancing osteogenesis and inhibiting osteoclastogenesis. While its mechanical properties are less suited to standalone implants, Sr’s incorporation into bone cement and scaffold coatings significantly improves their biological activity and the material’s stability. This makes Sr-based materials particularly effective in promoting bone regeneration in non-load-bearing applications or as coatings to enhance the performance of other metals.

In summary, the choice of metal depends on the specific clinical demands: magnesium is preferred for load-bearing applications, zinc for angiogenesis and immune modulation, and strontium for regulating bone metabolism and promoting regeneration. These complementary strengths provide a robust foundation for tailoring treatments to address the diverse challenges of osteoporotic fracture repair.

## 6. Challenges and Limitations

Despite the great potential of metallic materials in osteoporotic fracture repair, several challenges remain to be addressed.

First, high doses of metal ions may induce toxic reactions, particularly with their prolonged use, leading to tissue damage and cellular dysfunction. For instance, excessive Zn^2+^ and Fe^3+^ can trigger oxidative stress, resulting in cell apoptosis and necrosis [61]. Additionally, Cu^2+^ and Mg^2+^ at certain concentrations may provoke immune responses, adversely affecting repair outcomes [90]. To address these concerns, future research should prioritize the development of strategies to modulate the ion release kinetics, such as coating metallic materials with biocompatible polymers or incorporating ion release inhibitors. Advanced computational modeling and in vitro simulation could also help predict toxicity thresholds and design materials that balance therapeutic efficacy with safety.

Second, the dynamic balance between metal degradation products and in vivo reactions has not yet been fully resolved. The varying degradation rates of different metallic materials can impact their long-term efficacy and stability. Mg alloys, for example, release hydrogen gas during degradation, which can accumulate locally and form gas bubbles, potentially compromising the stability of bone healing. The degradation products of Fe and Sr may react with calcium and phosphate ions in the body, altering local mineral deposition processes and thus affecting bone repair. Therefore, designing metals with controllable degradation rates while maintaining the biocompatibility of their degradation products is another key challenge in the current research. In addition to these challenges, competitive concepts such as metal–ceramic and polylactide–ceramic composites and coatings are increasingly being investigated to address the limitations of metallic materials in osteoporotic fracture repair. Metal–ceramic composites, such as magnesium–hydroxyapatite hybrids, combine the mechanical strength and degradation properties of metals with the bioactivity and osteoconductivity of ceramics, offering a promising approach to improving osseointegration and local bone regeneration [91]. In addition, recent studies have explored the development of mesoporous silica nanoparticles (MSNs) capable of releasing silicon ions, offering a promising strategy for bone defect repair. These nanoparticles can be delivered via minimally invasive procedures or integrated into MSN/polymer composite scaffolds, providing the dual benefits of ionic stimulation and therapeutic drug delivery. This innovative concept represents a new approach to constructing multifunctional biomaterial systems aimed at promoting bone tissue regeneration [92].

Moreover, the use of animal models in metallic material research still has limitations. Although small animal models are widely used to evaluate metallic materials, they often fail to fully simulate the human bone repair process, particularly in the context of complex osteoporotic environments. The performance of metallic materials in animal models may differ significantly from clinical outcomes. For example, rabbits have a significantly different bone density and tissue composition compared to those in humans, while fractures in mice heal more quickly, making them less suitable for modeling the complex human bone repair process [93]. Additionally, the existing animal models often fail to adequately consider bone repair in patients with common bone diseases such as osteoporosis and diabetes, which limits the clinical applicability of the research results [94]. Therefore, selecting appropriate animal models and advancing their clinical translation are major issues that need to be addressed.

In summary, the application of metallic materials in bone repair holds great potential, but it still faces challenges related to toxicity, biocompatibility, the gap between animal models and clinical translation, and interactions in complex bone environments. Addressing these issues requires a multifaceted approach that integrates material innovation, improved experimental models, and translational research efforts. These advancements are essential for unlocking the full potential of metallic materials in bone repair.

Specifically, material design must prioritize the development of alloys with controllable degradation rates and non-toxic byproducts to mitigate adverse effects like tissue damage and inflammation. For instance, incorporating bioactive coatings or nanoparticle doping could enhance biocompatibility and regulate ion release, thereby reducing the cytotoxicity of materials such as Mg, Zn, or Sr. Advanced manufacturing techniques, including additive manufacturing and surface modification technologies, should be leveraged to fabricate implants with tailored mechanical properties and biofunctional surfaces. Additionally, translational research should emphasize the integration of preclinical findings into clinical trials. This could include designing pilot studies to evaluate implant safety and efficacy in specific patient populations, such as those with severe osteoporosis or metabolic bone diseases. Establishing standardized protocols for testing metallic materials under clinically relevant conditions will also be crucial for ensuring the reproducibility and accelerating regulatory approval processes.

## 7. Conclusions

The application of Mg, Zn, and Sr in osteoporotic fracture repair has shown great promise in providing mechanical support, promoting osteogenesis, and accelerating healing. However, challenges remain, including the toxic effects of excessive metal ions, balancing degradation products with biological responses, and ensuring stability in complex osteoporotic environments. Additionally, the gap between animal studies and clinical application hinders translation into practice.

Future research should focus on developing multi-metallic synergistic materials to enhance biocompatibility and bone healing while addressing toxicity and stability concerns. Advancements in surface modification technologies and the design of metal–biocomposite materials are anticipated to further improve the functionality and clinical potential of these innovative solutions.

## Figures and Tables

**Table 1:** Application of magnesium in osteoporotic fractures.

Year	Implant Type	Classification	Components	Method	Animal Species	Effects	Ref. *
2021	Mg	Base-material	Mg; bisphosphonates	One-step electrodeposition synthesis	In vitro	Enhanced proliferation and osteogenic differentiation of osteoblasts; no significant inhibition of osteoclasts	[10]
2021	Mg	Base-material	Mg; phytic acid; zoledronic acid	Facile one-step dip-coating method	In vitro	Promotion and suppression of the growth of pre-osteoblasts and osteoclasts	[49]
2023	Sr; Mg	Base-material	Mg; Sr hydroxyapatite	Electrodeposition technique	SD rats *	Exhibited a well-mineralized structure and good mechanical properties	[50]
2022	Mg	Base-material	Mg; melatonin; poly (lactic-co-glycolic acid)	Micro-arc oxidation and silane coupling	Castrated rats	Good cell viability, relative mRNA expression, and calcium deposition	[51]
2023	Mg; Nd; Zn; Zr	Base-material	Mg; Nd; Zn; Zr; zoledronic acid	3D printing	In vitro	Promoted osteogenic differentiation of bone marrow mesenchymal stem cells and inhibited the formation of osteoclasts and bone resorption	[61]
2024	Mg	Base-material	Mg; layered double hydroxide; graphene oxide quantum dots	One-step pulsed composite electrodeposition	OVX rats *	Promoted cell adhesion, migration, and differentiation; improved the local acidic environment	[62]
2024	Mg; Sr	Base-material	Mg; SrHPO_4_	Mixed solution stand for 24 h	SD rats *	Demonstrated therapeutic potential for alleviating postoperative pain by inhibiting osteoclast differentiation and enhancing the secretion of AICAR to suppress sensory innervation	[53]
2023	Mg	Admixture	Tetracalcium phosphate; Whitlockite; Mg	Wet precipitation method	Balb/c mouse	Good biocompatibility and osteoconductivity and better histocompatibility with the surrounding bone tissue	[54]
2023	Mg	Admixture	Magnesium oxychloride cement	Pickering foaming technique	In vitro	In vitro osteogenesis was significantly enhanced	[55]
2023	Mg	Admixture	Magnesium chloride; calcium sulphate; phosphate cement	Wet precipitation method	OVX rats *	Fast release of Mg provided a better outcome in terms of wound healing	[63]
2024	Mg	Admixture	Hydroxyapatite;Mg	Catalyzing sol–gel reaction	In vitro	Enhanced the osteogenic differentiation of BMSCs and induced macrophage polarization towards the M2 phenotype	[64]
2024	Mg	Admixture	Chitosan and teriparatide solution; magnesium phosphate cement	Homogeneous blending	In vitro	Promoted osteogenic differentiation and mineralization	[56]
2023	Mg; Zn; Ti	Coatings	Ti6Al4V; bisphosphonates; Mg; Zn	In situ hydrothermal crystallization	In vitro	Successful release of risedronate from the materials at a low level	[57]
2022	Mg	Coatings	Mg; Ga; Ti	Hydrothermal reaction	OVX rats *	Promoted autophagic activity and induced osteogenic differentiation of the mesenchymal stem cells (MSCs) while suppressing osteoclast generation	[58]
2022	Mg; Ti	Coatings	Ti6Al4V; icariin; Mg	3D printing	SD rats *	Improved the osseointegration between the PT and osteoporotic bone	[59]
2024	Mg	Coatings	Magnesium chloride; gallic acid	Stirring under reflux conditions	In vitro	Promoted osteogenic activity in RBMSCs and enhanced the angiogenic potential in HUVECs	[60]

* Ref.: reference; OVX rats: ovariectomized rats; SD rats: Sprague-Dawley rats.

**Table 2:** Application of strontium in osteoporotic fractures.

Year	Implant Type	Classification	Components	Method	Animal Species	Effects	Ref. *
2020	Sr	Admixture	Calcium phosphate silicate bioactive ceramic	Sol–gel method	Ovariectomy model	Sr-CPS promoted the osteogenic differentiation of rBMSCs through the Wnt/β-catenin signaling pathway and suppressed RANKL-induced osteoclast genesis through the NF-κB pathway	[75]
2020	Sr	Admixture	Calcium; strontium phosphates	Mixing and stirring precipitation	SD rats *	Increased the bone-volume-to-tissue-volume ratio, increased bone morphogenetic protein-2 (BMP2) expression, and significantly reduced the receptor activator of nuclear factor kappa-B ligand (RANKL)/osteoprotegerin (OPG) ratio	[80]
2020	Sr	Admixture	Sr; Ca; Si	Straightforward one-step method	SD rats *	Enhanced the expression level of osteogenic and angiogenic markers of osteoporotic bone mesenchymal stem cells (OVX BMSCs)	[74]
2021	Sr	Admixture	Sr; mesoporous bioactive glasses	Base-catalyzed sol–gel synthesis; aerosol-assisted spray-drying method	In vitro	Induced a decrease in bone resorption activity	[81]
2021	Sr	Admixture	Sr-doped nanoscale glass particles	Mixing and stirring precipitation	SD rats *	Profound bone regenerative potential in the cortical and surrounding trabecular area, including increased bone volume and density, the enhanced production of osteopromotive proteins, and more populated osteoblasts	[76]
2021	Sr	Admixture	Sr; bioactive glass	Mixing and stirring precipitation	Wistar rats	Improved the osteogenic potential of BMMSCs from rats treated with the bioactive glass extracts	[82]
2022	Sr	Admixture	Strontium-containing mesoporous glass particles	Mixing and stirring precipitation	In vitro	Sustained release of ICOS-Fc and Sr2+ ions for up to 28 days	[9]
2022	Sr	Admixture	SrCO3; poly (lactic-co-glycolic acid)	Freeze-grinding, mixing, and drying	In vitro	Promoted the proliferation of human fetal osteoblasts (hFOBs)	[83]
2020	Sr	Admixture	Sr; hydroxyapatite bioceramics	Mixing and stirring precipitation	SD rats *	Promoted the angiogenesis process and osteogenic activity	[84]
2020	Sr	Coatings	Strontium ranelate; POFC/β-TCP	3D printing	In vitro	Promoted the proliferation and differentiation of osteoblasts and guided bone regeneration	[77]
2020	Sr	Coatings	Polyetherketoneketonel; strontium	Chemical precipitation method	SD rats *	Promoted osteoporotic bone regeneration and delayed adjacent bone loss via regulating both osteoblasts and osteoclasts	[78]
2021	Sr	Coatings	Ginsenoside Rg1; SrHPO4; silk fibroin–gelatin	Chemical precipitation method	Ovariectomy model	Promoted osteogenesis and angiogenesis	[85]
2022	Sr; Mg	Coatings	Strontium-doped hydroxyapatite	Electrodeposition technique	Ovariectomy model	A large amount of bony tissue with higher trabecular thickness and hardness was formed	[50]
2022	Sr; Ti	Coatings	Sr; Ti; bioactive glass particles	Chemical precipitation method	Ovariectomy model	Positive effect on the formation of new bone	[79]
2022	Sr; Ce; Ti	Coatings	Sr; Ce; Ti;	Hydrothermal procedure	SD rats *	Restored MSC function in the implant site and promoted new bone formation	[86]
2022	Sr	Coatings	Strontium carbonate	Electrodeposition method	In vitro	Inhibitory effect on bacterial growth in both Gram-positive and Gram-negative strains	[87]
2023	Sr; Ti	Coatings	Sr; Ti	Stirring, dissolution, annealing, and solidification	In vitro	Strong osteogenic effect	[88]

* Ref.: reference; OVX rats: ovariectomized rats; SD rats: Sprague-Dawley rats.

## Data Availability

The datasets used and/or analyzed during the current study are available from the corresponding author on reasonable request.
